# BAG3: a new player in the heart failure paradigm

**DOI:** 10.1007/s10741-015-9487-6

**Published:** 2015-04-30

**Authors:** Tijana Knezevic, Valerie D. Myers, Jennifer Gordon, Douglas G. Tilley, Thomas E. Sharp, JuFang Wang, Kamel Khalili, Joseph Y. Cheung, Arthur M. Feldman

**Affiliations:** Department of Neuroscience, Temple University School of Medicine, 3500 N. Broad Street, Suite 1150, Philadelphia, PA 19140 USA; Department of Physiology, Temple University School of Medicine, 3500 N. Broad Street, Suite 1150, Philadelphia, PA 19140 USA; Department of Pharmacology, Temple University School of Medicine, 3500 N. Broad Street, Suite 1150, Philadelphia, PA 19140 USA; Department of Medicine, Temple University School of Medicine, 3500 N. Broad Street, Suite 1150, Philadelphia, PA 19140 USA

**Keywords:** BAG3, Apoptosis, Autophagy

## Abstract

BAG3 is a cellular protein that is expressed predominantly in skeletal and cardiac muscle but can also be found in the brain and in the peripheral nervous system. BAG3 functions in the cell include: serving as a co-chaperone with members of the heat-shock protein family of proteins to facilitate the removal of misfolded and degraded proteins, inhibiting apoptosis by interacting with Bcl2 and maintaining the structural integrity of the Z-disk in muscle by binding with CapZ. The importance of BAG3 in the homeostasis of myocytes and its role in the development of heart failure was evidenced by the finding that single allelic mutations in BAG3 were associated with familial dilated cardiomyopathy. Furthermore, significant decreases in the level of BAG3 have been found in end-stage failing human heart and in animal models of heart failure including mice with heart failure secondary to trans-aortic banding and in pigs after myocardial infarction. Thus, it becomes relevant to understand the cellular biology and molecular regulation of BAG3 expression in order to design new therapies for the treatment of patients with both hereditary and non-hereditary forms of dilated cardiomyopathy.

## Introduction

**Fig. 1:**
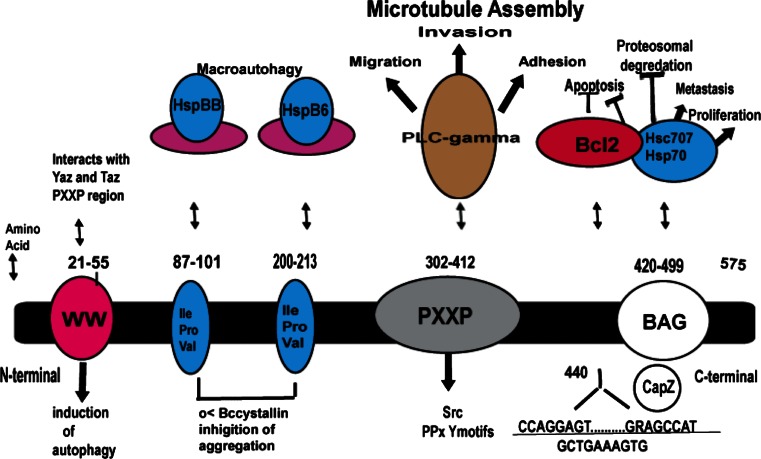
BAG3 protein adapted from McCollum et al. [118]

**Fig. 2:**
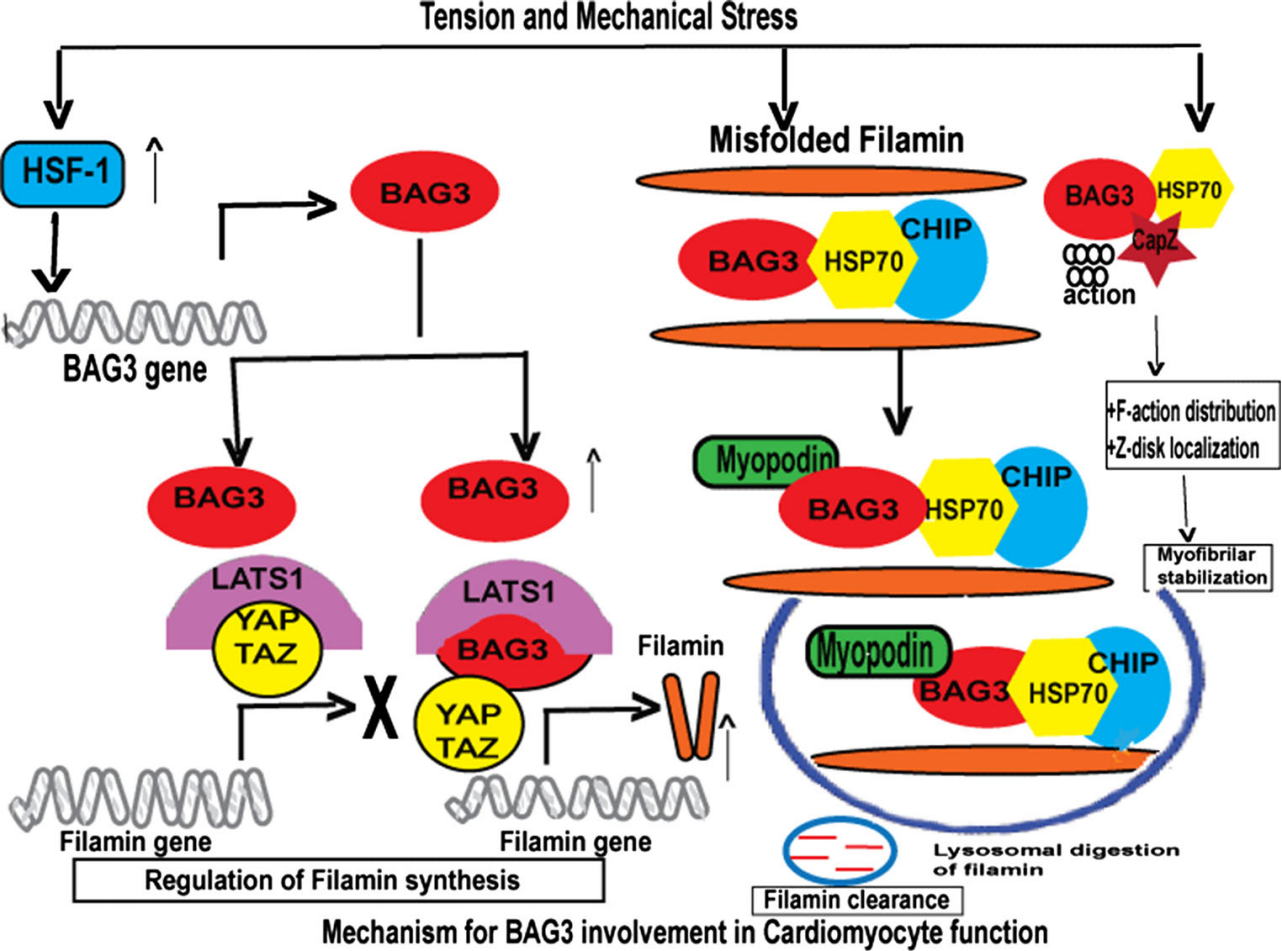
Role of BAG3 in the cell

Bcl2-associated athanogene 3 (BAG3) is a 575 amino acid anti-apoptotic protein that is constitutively expressed in the heart, skeletal muscle and some cancers and serves as a co-chaperone of both the constitutively and non-constitutively expressed heat-shock proteins (Hsc/Hsp) [1, 2] (Fig. 1). When bound to Hsp’s, BAG3 plays a critical function in regulating protein quality control (PQC) [2] and by interacting with Bcl2, it protects cells from apoptotic death [3]. The BAG3-HSP protein–protein interaction is increasingly recognized as a therapeutic target in the treatment of cancer [4, 5]. Recently, it has been shown that BAG3 plays a role in the stability of the cardiac sarcomere through regulation of filamin clearance and production and by binding to CapZ [6] (Fig. 2). Two seminal findings led to the recognition that BAG3 could play a substantive role in the development of or progression of heart failure. First, Homma showed that mice with homozygous disruption of BAG3 developed a fulminant myopathy characterized by non-inflammatory myofibrillar degeneration, disruption of Z-disk architecture, apoptotic features in the early postnatal period and death by 4 weeks of age [7]. Second, Selcen reported three children with myofibrillar myopathy who harbored a single allelic substitution of BAG3 [8]. In addition, knockdown of BAG3 in zebrafish [9] or in neonatal cardiomyocytes [10] leads to significant cardiac dysfunction. Thus, BAG3 appears to be an exciting new target for therapeutic intervention in patients with heart failure. Here, we review our current understanding of the biology and pathobiology of BAG3 as it relates to the heart.

## BAG3 mutants, myofibrillar myopathy and dilated cardiomyopathy

The first evidence that BAG3 could play an important role in the pathobiology of the heart came from a study by Homma which demonstrated that mice in which BAG3 had been knocked out had non-inflammatory myofibrillar degeneration, disruption of Z-disk architecture, apoptotic features in the early postnatal period and death by 4 weeks of age [7]. However, it was the finding that mutations in BAG3 were associated with the development of muscle disease in children that led investigators to propose that changes in BAG3 function could result in the development of left ventricular dysfunction and heart failure. Selcen and co-workers first reported three children with myofibrillar myopathy who harbored a single allelic substitution of Leucine (Leu) for Proline (Pro) at position 209 (exon 3 of BAG3) of BAG3 [8]. All three patients presented in childhood with progressive muscle weakness, respiratory insufficiency and cardiac dilatation with systolic dysfunction. The parents were asymptomatic and did not have the Pro209Leu genotype. Odgerel et al. [11] reported an additional three families with the same BAG3 p.Pro209Leu genotype, but the severe myofibrillar myopathy in these patients was accompanied by axonal neuropathy with giant axons. One asymptomatic parent showed somatic mosaicism, whereas in the other two families, the parents had a normal genotype supporting the observation by Selcen that spontaneous mutations could occur. In this group of patients, early respiratory failure was more common than heart failure [12].

The first suggestion that BAG3 could play a role in adult-onset familial dilated cardiomyopathy came from a study of patients with a dilated cardiomyopathy, diffuse myocardial fibrosis and sudden death. The phenotype was associated with a locus on chromosome 10q2-26, a region that included the BAG3 gene [13]. Two mutations in BAG3 were subsequently identified in Japanese patients with familial dilated cardiomyopathy (Arg218Trp and Leu462Pro). When these mutations were expressed in neonatal rat cardiomyocytes, functional studies showed impaired Z-disk assembly and increased sensitivity to stress-induced apoptosis [14]. Norton et al. [9] identified a deletion of BAG3 exon 4 as causative of familial dilated cardiomyopathy in a family without neuropathy or peripheral muscle weakness. Zebrafish expressing this mutation demonstrated cardiac enlargement and hypertrophy. Subsequent sequencing of BAG3 in subjects diagnosed with idiopathic dilated cardiomyopathy (IDC) identified four additional mutations that segregated with all relatives affected by the disease. A genome-wide association study (GWAS) conducted in patients with HF secondary to IDC implicated a non-synonymous single nucleotide polymorphism (SNP) (c.757T > C, [p. Cys151Arg]) located within the BAG3 gene as contributing to sporadic dilated cardiomyopathy [15]. More recently, we found a 10 nucleotide mutation in exon 6 of the BAG3 gene in a large family with familial dilated cardiomyopathy [16]. The mutation segregated with all affected family members and predicted a shift in the reading frame that would result in the deletion of 135 amino acids from the C-terminal end of the protein that encompassed a large portion of the BAG region [16].

**Fig. 3:**
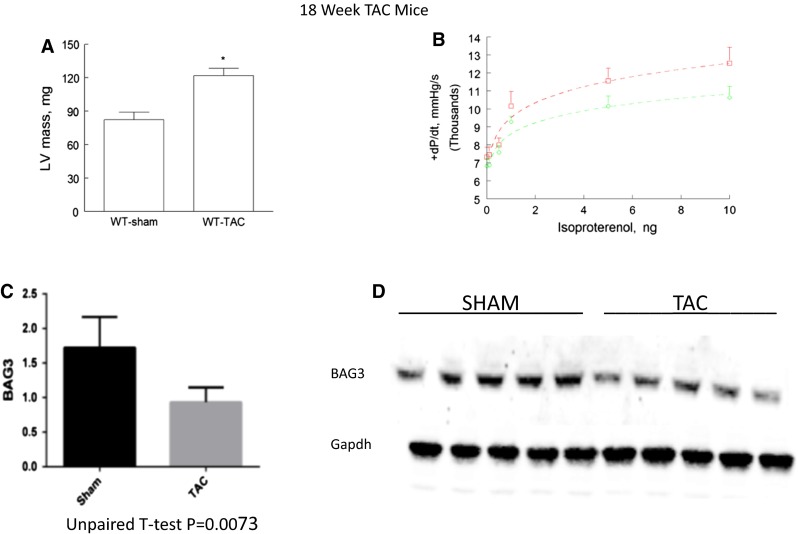
BAG3 levels in failing murine hearts. Wild-type c57BL/6 mice underwent trans-aortic banding (TAC) as has been described previously [119]. Eighteen weeks after TAC, *left ventricular* contractility was measured using a conductance catheter inserted into the *left ventricle* through a carotid approach as described previously. Heart weight to body weight ratios were calculated after killing (**a**). Contractility was measured during intravenous infusion of increasing doses of catecholamine (**b**). **b** Sham-operated hatched line = control; solid line = TAC mice. Hearts were then frozen for subsequent measurement of BAG3 levels. Myocardial proteins were extracted as we have described previously separated by gel electrophoresis and probed with a murine BAG3 antibody. As seen in **c**, there was a significant decrease in BAG3 levels by western blotting in TAC mice when compared with sham-operated controls. A representative western blot is seen in **d**

**Fig. 4:**
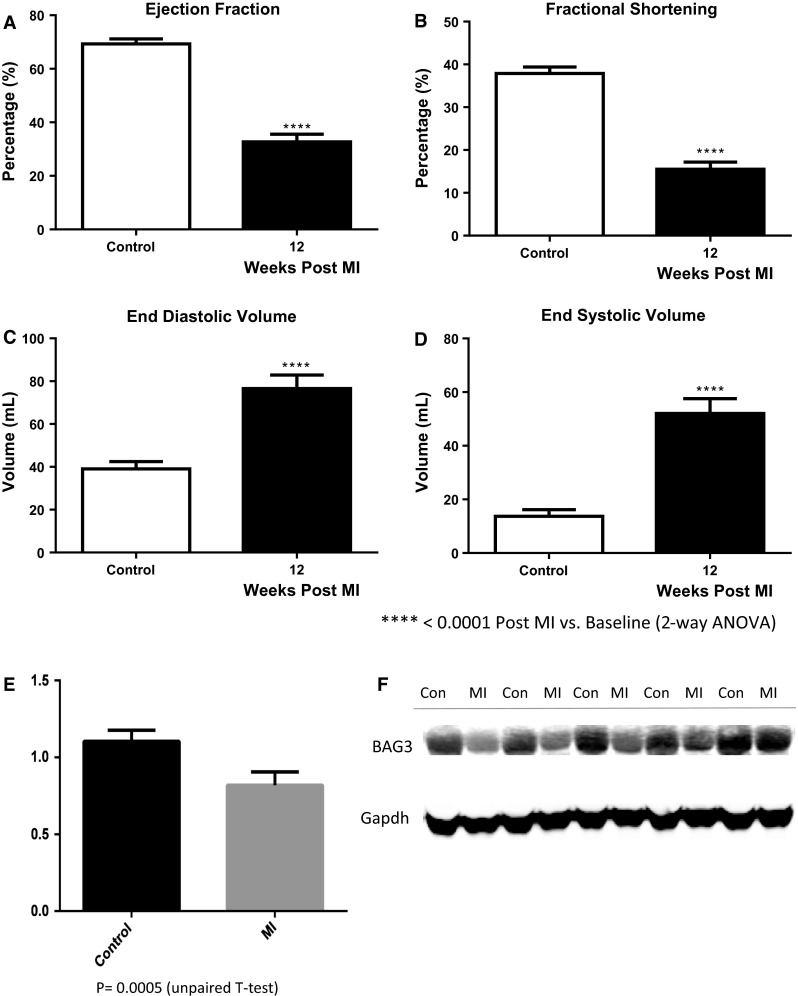
Hemodynamic indices and BAG3 levels in non-infarcted *left ventricular* myocardium from a pig 4 weeks after balloon occlusion of the *left anterior* descending coronary artery. **a** ejection fraction; **b** fractional shortening; **c** end diastolic volume; **d** end systolic volume; **e** BAG3 levels; **f** representative western blot

Interestingly, a western blot of protein extracted from the left ventricular myocardium of a family member who underwent heart transplantation demonstrated a level of BAG3 in the heart that was less than half of that seen in non-failing control hearts obtained from organ donors whose hearts could not be used for transplant because of blood type or size incompatibility. However, our finding that levels of BAG3 were also diminished by nearly 50 % in hearts from patients with end-stage heart failure undergoing cardiac transplantation that had a normal BAG3 genotype led us to propose that deficiencies in BAG3 might be a critical component in the progression of heart failure humans [16]. Indeed, as seen in Fig. 3, we have also found that mice with severe heart failure 18 weeks after trans-aortic banding demonstrate significant decreases in BAG3 levels that are comparable to the decrease seen in patients with heart failure. Similarly, pigs with HF secondary to occlusion of the left anterior descending coronary artery (Fig. 4a–d) also demonstrated significant reductions in levels of BAG3 (Fig. 4e, f). The decrease in BAG3 in humans, pigs and mice with heart failure was not associated with a change in the levels of BAG3 mRNA suggesting that posttranslational modifications account for the decrease. A recent report demonstrates that BAG3 levels are increased in the sera of patients with HF [17]. The same group also reported increased levels of BAG3 antibodies in the sera of patients with HF [18]. However, by contrast with most biomarkers including BNP and TNFx [19], BAG3 levels were only decreased in patients with NYHA Class IV HF, although these results will need to be confirmed in a larger group of patients.

Genetic heterogeneity is a common feature of genetic mutations in cardiac genes, and thus, it is not surprising that individuals with mutations in BAG3 can present with a variety of cardiovascular phenotypes. For example, in the family that we reported, the onset of symptomatic heart failure occurred as early as 18 years of age and as late as 48 years of age. BAG3 mutations can also be associated with a diverse set of phenotypes. For example, a Chinese patient with restrictive lung disease, a rapidly progressive proximal myopathy, rigid spine, bilateral Achilles tendon tightening, hypertrophic cardiomyopathy with restrictive physiology and a prolonged QT interval had de novo mutation at c.626C > T (p.Pro209Leu) which is situated in the conserved Ile-Pro-Val motif that is a site of interaction between BAG3 and the Hsps as well as a second non-synonymous change c.772C > T (p.Arg258Trp) which was not situated in a known structural domain [20]. Interestingly, polymorphisms of BAG3 may also play a role in the pathogenesis of tako-tsubo cardiomyopathy [21].

### Structure–function relationships of BAG3

A partial sequence of BAG3 protein was first isolated in 1999 using a yeast two-hybrid screen with Hsp70 as bait [22] (Fig. 1). Investigators cloned BAG3 from cDNA libraries using recombinant Bcl2 [23, 24]. BAG3 is highly conserved in nature at both the gene and protein level with significant homology across mice, pigs and humans [25]. By contrast, BAG3 has little in common with the other members of the BAG family with the exception that all members of the family share a common BAG domain. Located at the C-terminal end of the protein, this region consists of three alpha helices of 30–40 amino acids each that bind to a motif in the ATPase domain of Hsp70, to Bcl2 and to small heat-shock proteins (HspX or sHsp) [26]. The length of the BAG domains varies with two distinct forms: A ‘long’ BAG domain that is specific for BAG1 and a ‘short’ domain that is found in BAG3, BAG4 and BAG5 [27]. Only BAG1 and BAG3 interact with Bcl2, and BAG4 is physiologically distinguishable from the other BAG family members in that it blocks TNF receptor signaling [24, 28–32]. BAG6 (Scythe) regulates nuclear pathways and cytochrome c release [33, 34]. BAG3 also contains a WW domain near its N-terminal region [35] and a PXXP domain that binds phospholipase Cγ-1 [36]. The WW domain and the PXXP domain may also connect BAG3 to the SH3 domain of Src thereby mediating the effects of Hsp70 on Src signaling and to PPxY motifs of signaling proteins providing a platform for the assembly of multi-protein networks [37]. BAG3 also binds to αB-crystallin via a highly conserved intermediate domain (Ile-Pro-Val) that facilitates its ability to inhibit protein aggregation [38].

Studies using Htt43Q, a pathogenic form of huntingtin that is responsible for Huntington’s disease, as a molecular probe have helped to define the role of some of the motifs found in BAG3. The BAG domain is required for interaction with Hsp70 and Bcl-2 but not with HspB8 (HspB8 and HspB6 bind to IPV domains) yet BAG3 is able to clear huntingtin even in the absence of the BAG region [39, 40]. By contrast, deletion of the WW domain had no effect on Hsp70, Bcl-2 or HspB8 binding and had no effect on Htt43Q degradation. Deletion of the proline-rich PXXP region also did not alter Hsp70, Bcl-2 or HspB8 binding—but abrogated the ability of the cell to clear Htt43Q [40]. The only protein known to interact with the PXXP proline-rich region of BAG3 is phospholipase Cγ-1 which modulates microtubule assembly [36, 41]. However, PKCγ-1 knockdown had no affect on the ability of BAG3 to clear Htt43Q. Taken together, these results suggest that HspB8 plays an important role in the clearance of mutated proteins such as Htt43Q; however, the specific role of the PXXP region remains to be defined.

### Regulation of BAG3 expression

BAG3 expression is increased by the stress associated with heavy metals, high temperature [42, 43] oxidants [44], proteasome inhibitors [45], serum starvation [46] light damage in the retina [47]; seizure activity [48]; hemodialysis [49]; hypoxia [50]; and HIV infection [51]. In addition, BAG3 expression is increased in a large number of cancers including: acute lymphocytic and B cell chronic lymphocytic leukemia [52, 53] thyroid carcinoma [54]; melanomas [55] non-small-cell lung cancer [56]; hepatocellular carcinoma [57]; pancreatic adenocarcinoma [58], small cell carcinoma of the lung [59] and glioblastoma [60]. The overexpression of BAG3 in malignant cells increased motility and metastasis, whereas reduction in BAG3 protein by RNA interference decreased cell motility. Cells from BAG3-deficient mice showed delayed formation of filopodia and focal adhesion complexes—putatively mediated by decreased activity of the small GTPase Rac1 that is involved in actin cytoskeleton dynamics. Furthermore, mice with reduced BAG3 showed suppressed invasion and metastasis of a human tumor xenograft [61]. Consistent with the finding that BAG3 is involved in cell adhesion, motility and metastasis of cancer cells, Franco et al. [55] demonstrated that melanoma tumors that have metastasized to distant organs had high levels of BAG3 expression. Interestingly, BAG3 is able to modulate its own transcription through a positive feedback loop involving its 5′-untranslated region (UTR) sequence—a process that is mediated by the BAG domain but is independent of BAG3 association with the UTR sequence [62]. This ability to self-regulate in a positive manner may account for the long-term survival of malignant cells.

The predominant mechanism through which stress increases BAG3 expression is induction of heat-shock factor 1 (HSF1) [63]. Stress causes induction of both HSF1 and the HSF target gene DNAJB1 in smooth muscle although the predominant factors regulating BAG3 expression in the heart have not been defined [64]. WT1, an oncogene that is expressed in a variety of tumors and that is associated with a poor response to therapy also induces BAG3 expression by binding to sequences in the promoter region of BAG3 [65, 66]. By contrast, serum starvation downregulates BAG3 expression at the transcriptional level via c-Jun [46]. BAG3 expression is also regulated by the transcription factors Egr1 and AibZIP [67, 68]. Importantly, BAG3 function and levels can also be regulated by posttranslational modification. For example, phosphorylation of Ser187 of BAG3 by protein kinase C delta (PKCΔ) leads to increased epithelial-mesenchymal transition, motility and invasiveness of cancer cells [69, 70]. Expression of exogenous Tat, a protein expressed by the HIV virus, in glioblastoma cell lines enhances BAG3 protein but not mRNA levels [71]. BAG3 levels are also increased by drugs including: TNF-related apoptosis-inducing ligand, fludarabine, cytosine arabinoside and etoposide [1, 45, 54, 72]. Proteasome inhibitors induce a BAG3-dependent non-canonical autophagy in HepG2 cells although the specific mechanism for this effect has not been elucidated [73]. BAG3 levels are also enhanced by decreased calcium influx as caused by exposure to carboxyamido-triazole (CAI) an inhibitor of non-voltage-gated calcium channels [36]. The physiologic significance of this finding in cardiomyocytes is under investigation in our own laboratories. Both JNK and NF-kB induce BAG3 expression in the presence of lipopolysaccharide suggesting that BAG3 is also responsive to the stress associated with enhanced expression of pro-inflammatory cytokines and therefore may participate in inflammatory diseases including that of the heart [74]. The p38 MAP kinase increases BAG3 transcription in HeLa cells exposed to oxidative stress, although the presence of this pathway in cardiac cells has not be elucidated [75].

## Autophagy and apoptosis

All eukaryotic cells depend on the presence of a system for protein quality control (PQC). PQC acts as a surveillance system that assures proper protein folding as well as recognition of misfolded and dysfunctional proteins or protein aggregates and initiates protein refolding or clearance. BAG3 plays a critical role in this process (Fig. 2). PQC relies on molecular chaperons and co-chaperons that can sense misfolded proteins and then either initiate refolding or elimination of the folded or damaged proteins from the cell. Eukaryotic cells have two major intracellular protein degradation pathways, ubiquitin–proteasome and autophagy-lysosome systems. The ubiquitin–proteasome system (UPS) is composed of a barrel-shaped protein complex with a 13-A wide opening through which ubiquinated misfolded proteins have to pass in order to be degraded into smaller reusable peptides [76]. Some protein aggregates are too large to fit into the 13-A wide channel of the proteasome. These large aggregates are degraded by the aggresome-autophagy system. Chaperones and co-chaperones can participate in both of these systems by identifying selective proteins for destruction. In fact, the BAG family of proteins can regulate whether misfolded proteins are degraded by the proteasomal or by the autophagy pathways. Autophagy systems can be divided into macroautophagy, microautophagy and chaperone-mediated autophagy [77] (Figs. 1, 2).

In a multi-step process, macroautophagy sequesters protein aggregates in autophagasomes, double-layered membrane structures found in the cytoplasm. The protein aggregates are then transported to and fused with the lysosome for degradation by lysosomal hydrolases [78]. In microautophagy, the cargo enters lysosomes directly by invagination of the lysosomal membrane resulting in degradation of the aggregated protein content by lysosomal enzymes. Macroautophagy is a somewhat promiscuous system; however, recent studies have shown that BAG3 also participates in selective macroautophagy that is responsible for homeostatic regulation of specific proteins. When these clearance mechanisms become overwhelmed in neuronal cells, increased levels of abnormal protein aggregates can lead to the progression of a number of neurologic diseases including Alzheimer’s disease, Parkinson’s disease, Huntington’s disease and spinocerebellar ataxia type 3 [79–82]. Indeed, overexpression of BAG3 induced decreases in tau, a protein that plays a fundamental role in the pathogenesis of Alzheimer’s disease [83].

In selective macroautophagy, BAG3 is coupled with the chaperone Hsp70 and the co-chaperone ubiquitin ligase carboxyl terminal of Hsp70/Hsp90 interacting protein (CHIP) and facilitates the sequestration of misfolded proteins into autophagasomes [84]. Investigators have recently focused their interest on two IPV (Ile-Pro-Val) motifs in BAG3 that regulate its stoichiometric interaction with the small heat-shock proteins (sHsp) sHsp6 and sHsp8 [40, 85, 86]. The multi-chaperone complex of BAG3-HspB8-Hsp70 can selectively cause misfolded proteins to be degraded by macroautophagy—a function that may require the cooperation of the macroautophagy receptor protein p62/SQSTMI. These proteins in concert can bind simultaneously to ubiquitin and the autophagasome membrane-associated protein LC3 [85, 87–90]. Once coupled to the chaperone and co-chaperone complexes, misfolded proteins as well as autophagic vacuoles are transported retrograde along cytoskeletal tracks by ATP-fueled motor proteins called dynein motor proteins to perinuclear microtubule organizing centers or MTO’s. Once there, they are packaged in protein structures called aggresomes for eventual disposal or engulfed and degraded by the autophagic vacuoles [91–94]. Recently, Gamerdinger and colleagues have reported that BAG3 mediates the transport of proteins to the aggresome by catalyzing substrate transfer from Hsp70 to the dynein motor complex [78, 89].

A second major role for BAG3 is that it inhibits apoptosis through multiple mechanisms—many of which have been elucidated in cancer cells. Multiple forms of cellular stress and noxious stimuli activate signals that converge into a common pathway that is triggered by caspases [95–97]. The anti-apoptotic members of the Bcl-2 family of proteins (Bcl-2 and Bcl-x) inhibit caspase activation by blocking the release of apoptogenic cytochrome c from the mitochondria and by sequestering the procaspases 8 and 9 [98–104]. BAG3 synergizes with Bcl-2 and with Bcl-XL to protect both normal cells and neoplastic cells from apoptosis [44, 53, 56]. Its overexpression can synergize the anti-apoptotic effect of Bcl-2 [24], whereas BAG3 knockdown increases both basal and drug-induced apoptosis.

BAG3 overexpression can also inhibit apoptosis by modulating the NF-kB pathway [105]. BAG3 mediates the dissociation of the Hsp70- kappa B kinase (IKK-γ: subunit of IKK) complex, which leads to a decrease in Hsp70-mediated delivery of IKK-γ to the proteasome thereby sustaining NF-kB activation and inhibition of cell apoptosis [55]. However, investigators have also reported that NF-kB can modulate the expression of BAG3 as well as the formation of the BAG3-HsB8 complex [106]. Recent studies have suggested a broader role for BAG3. For example, BAG3 regulates epithelial-mesenchymal transition and angiogenesis through ERK phosphorylation [57, 107]; induces epithelial-mesenchymal transition through activation of the transcription factor ZEB1 [108]; and modulates the activity of the transcription factors FoxM1 and Hif1α, the translation regulator HuR and the cell cycle regulators p21 and survivin [109]. BAG3 also downregulates the microRNA-29b which leads to upregulation of the anti-apoptosis protein Mcl-l leading to resistance to anticancer drugs [110].

### The molecular mechanisms by which BAG3 modulates the cardiac phenotype

The finding—that mutations in BAG3 were associated with the development of disrupted Z-disk structure, myofibrillar degeneration and disorganization—led Hishiya and colleagues to assess the effects of BAG3 in neonatal rat myocytes [8, 10, 111]. They found that BAG3 insured the structural stability of filamentous actin (F-actin) by promoting association between Hsc70 and the actin capping protein beta 1(CapZβ1). BAG3 also facilitated the cellular localization of CapZβ1. CapZβ1 is a sarcomere protein that: (1) binds with high affinity to the barbed end of actin to prevent its disassociation into actin monomers; (2) interacts with the protein nebulin to position the actin filaments at the Z-disk; (3) links adjacent sarcomeres; and (4) stabilizes the Z-disk [112–114]. BAG3 knockdown led to proteasomal degradation of CapZβ1, whereas inhibition of CapZβ1 led to myofibril disruption in response to mechanical stress. By contrast, overexpression of CapZβ1 prevented myofibril disruption when BAG3 was knocked down [10]. These results were consistent with the finding that mutations in many of the genes encoding Z-disk proteins lead to increased vulnerability to mechanical stress [115, 116], For example, mutations in sarcomere genes such as desmin, αB-crystallin, myotilin, Z-band alternatively spliced PDZ motif containing protein (ZASP) and filamin C result in phenotypes that are very similar to that seen in cells in which BAG3 has been knocked down. Thus, the co-chaperone BAG3 and the chaperone Hsc70 play a critical role in maintaining the structural integrity of the sarcomere especially during mechanical stress.

Recent studies have shown that BAG3 can also modulate the level of functional filamin, a dimeric actin cross-linker that acts as a signaling hub for various proteins and that also plays an important role in stabilization of the myofibrillar Z-disk [117]. BAG3 removes filamin that has been damaged by mechanical stress through autophagic mechanisms. At the same time, BAG3 stimulates filamin transcription by using its WW domain to engage inhibitors of the transcriptional activators YAP and TAZ [64].

Filamin regulation and clearance and sarcomere stabilization appear to occur in large part through what is now referred to as chaperone-assisted selective autophagy or CASA [6]. CASA differs from macroautophagy described above in that it requires a multi-chaperone complex comprised of a client protein, HspA8-/Hsp70-, HspB8-/Hsp27- and the HspA8-associated ubiquitin ligase STUB/CHIP as well as ubiquitin conjugation enzymes of the UBE2D family. The degradation signal generated by ubiquinitation of the client protein leads to recognition by the autophagic ubiquitin adaptor SQSTMI/p62, autophagasome formation, and protein degradation in lysosomes. This pathway is relevant to muscle as the CASA machinery is localized at the Z-disk, and CASA knockdown leads to disintegration of the Z-disk and resultant pathologic changes in skeletal and cardiac muscle. A predominant feature of CASA is that it is stress related with a predominant substrate of CASA in mechanically stressed cells being the cytoskeletal protein filamin. Thus, in the heart, BAG3 helps rid the myocyte of misfolded and degraded proteins but also maintains the homeostatic balance between filamin breakdown and filamin production.

In adult mouse left ventricular myocytes in which endogenous BAG3 is knocked down by adenovirus-siRNA, we recently observed that systolic calcium concentrations, calcium transient amplitude and single myocyte contraction amplitude are all significantly decreased compared to myocytes infected with adenovirus-GFP (unpublished results). These observations suggest that in addition to regulating CapZB1 and filamin, BAG3 may modulate cardiac contractility by affecting myocyte excitation–contraction coupling.

## Conclusion

In summary, BAG3 ‘chaperones’ an array of cellular proteins including the Hsps and the sHsps that play a critical role in maintaining the homeostasis of eukaryotic cells and the balance between autophagy and apoptosis. BAG3 is of particular importance during cell stress as increased apoptotic signals and aggregates of protein debris threaten cell survival. Appropriate levels of BAG3 production and function are of particular importance in the heart because the complex components of the sarcomere are continuously exposed to contractile stretch and strain leading to changes in protein folding and in apoptotic signaling. In addition, BAG3 through binding to CapZ helps to maintain the highly ordered filamentous structure of the Z-disk by clearing filament debris while at the same time stimulating filament synthesis. That BAG3 plays an important role in the progression of heart failure is demonstrated by the finding that loss of function mutations result in the development of both early-onset and late-onset familial dilated cardiomyopathy. However, additional research is required to: (1) elaborate the molecular and cellular mechanisms that account for the decrease in BAG3 levels seen in hearts from patients with end-stage heart failure; (2) identify the effects of cardiac stress and left ventricular dysfunction on the chaperone and co-chaperone peptides that partner with BAG3 including the Hsp’s, sHsp’s, myopodin and synaptopodin; and (3) assess whether reconstitution of normal levels of BAG3 alone can interrupt the progression of heart failure. Perhaps the most interesting question derives from the fact that while BAG3 expression maintains cell survival by inhibiting apoptosis and by removing the debris that accumulates in cells that are under continuous mechanical tension such as cardiac myocytes, these mechanisms are maladaptive in the presence of malignant cells as increased levels of BAG3 can decrease apoptosis leading to increased tumor growth, enhanced metastasis, decreased sensitivity to chemotherapeutic agents and reduced survival. With BAG3 serving as a new target for chemotherapy, additional studies will be needed to develop approaches that will enhance apoptosis and decrease autophagy in malignant cells while at the same time not influencing these pathways in the heart.
